# Biphasic calcium phosphate in periapical surgery

**DOI:** 10.4103/0972-0707.44059

**Published:** 2008

**Authors:** Chinni Suneelkumar, Krithika Datta, Manali R Srinivasan, Sampath T Kumar

**Affiliations:** Department of Conservative Dentistry and Endodontics, Narayana Dental College, Nellore, India; 1 Department of Conservative Dentistry and Endodontics, Meenakshi Ammal Dental College, Chennai, India; 2 Department of Conservative Dentistry and Endodontics, Sri Venkateswara Dental College, Chennai, India; 3 Department of Bioceramics, Indian Institute of Technology (Madras), Chennai, India

**Keywords:** Biphasic calcium phosphate, bone substitute, hydroxyapatite, osteoconductive, periapical surgery, β-tricalcium phosphate

## Abstract

Calcium phosphate ceramics like hydroxyapatite and β -tricalcium phosphate (β -TCP) possess mineral composition that closely resembles that of the bone. They can be good bone substitutes due to their excellent biocompatibility. Biphasic calcium phosphate is a bone substitute which is a mixture of hydroxyapatite and β -tricalcium phosphate in fixed ratios. Studies have demonstrated the osteoconductive potential of this composition. This paper highlights the clinical use of biphasic calcium phosphate as a bone substitute in periapical surgery.

## INTRODUCTION

Although nonsurgical endodontic treatment gives good results in most cases, surgery may be indicated for teeth with persistent pathoses that have not responded to nonsurgical approaches and teeth with large periradicular lesions greater than 15 mm in diameter. Surgical endodontics encompasses those surgical procedures performed to remove the causative agents of radicular and periradicular disease and to restore these tissues to functional health. After curetting the periapical lesion, the bony defect can be filled with a graft material to enhance the bone formation.[1]

Based on the type of bone graft material used, all graft materials can be categorized into four different categories:

- Autograft or autogenous bone graft
- Allograft or allogenic bone graft
- Xenograft or xenogenic bone graft
- Alloplast or alloplastic bone graft (synthetic)

The autograft is considered the gold standard. It is defined as tissue transplanted from one site to another within the same individual. It is basically the patient's own bone taken from a donor site and placed somewhere else in the body, into the recipient site. The best success rates in bone grafting have been achieved with autografts because these are essentially living tissues with their cells intact. There is no immune reaction and the microscopic architecture is perfectly matched. The only disadvantage of the autograft is that it has to be harvested from a secondary site in donor body, which usually means more morbidity and a more complicated surgery, overall. Sometimes, however, when there is not enough bone volume available intraorally, we have to get bone from other parts of the body.[2,3]

The allograft is defined as a tissue graft between individuals of the same species (i.e., humans) but of non-identical genetic composition. The xenograft is defined as a tissue graft between two different species (i.e., bone of bovine origin). The alloplast usually includes any synthetically derived graft material not (coming) from animal or human origin. This usually includes hydroxyapatite, β-TCP, or any formulation thereof. Each of the bone graft materials is usually developed with a specific purpose or advantage in mind. The main purpose of using the latter three of the above graft materials is usually to avoid a secondary surgery for harvesting autogenous bone.[2,3]

Intensive investigative research has shown calcium phosphate ceramic biomaterials to be safe and effective for a variety of restorative and preservative clinical applications. Two forms of calcium phosphate ceramics, hydroxyapatite (HA) and tricalcium phosphate (TCP), have received attention.[4] They possess a mineral composition very close to that of normal bone. Their biocompatibility makes them successful bone substitutes.

HA is the natural mineral component of vertebrate hard tissue, comprising 60% to 70% of bone and 98% dental enamel.[5] In appropriate synthetic form, HA is generally observed to be non-bioresorbable and therefore accountable for long-term restorative and preservative clinical procedures. TCP is clinically similar to HA but is not a natural component of bone. It is partially bioresorbable and is often considered desirable for repair of morphological site.

Calcium phosphate biomaterials are devoid of local or systemic toxicity, do not elicit inflammatory or foreign body responses, can become functionally integrated with natural bone with no fibrous tissue encapsulation and cause no generation of normal bone mineralization process.[6] Calcium phosphate biomaterial provides a physical matrix suitable for deposition of new bone and can display growth-guiding properties causing bone to extend its growth into areas that otherwise it would not occupy.[7] It also has the ability to maintain bone bulk in areas where bone resorption formally takes place. HA implants have been used successfully to prevent post-extraction alveolar ridge resorption.

Biphasic calcium phosphate ceramics consist of both β-tricalcium phosphate and hydroxyapatite. The alliance of these two components allows bioactivity to be controlled through a combination of HA and β-TCP properties.[8–10] Previous studies have shown that biphasic calcium phosphate (BCP) has osteoconductive potential. The development of biphasic calcium phosphate ceramic made it possible to control the resorbability of tricalcium phosphate and at the same time maintain the osteoconductive potential of hydroxyapatite. This paper highlights the use of BCP as bone graft material in endodontic periapical surgery.

## CASE REPORT

A 29-year-old female patient reported to the department with acute pain in right upper front tooth. Tooth #7 was discolored and was not responding to thermal and electric pulp tests. Radiograph revealed a large periapical lesion in relation to tooth #7 [Figure 1A]. Access preparation was done and canal was debrided with minimal cleaning and shaping of the root canal.

**Figure 1A:**
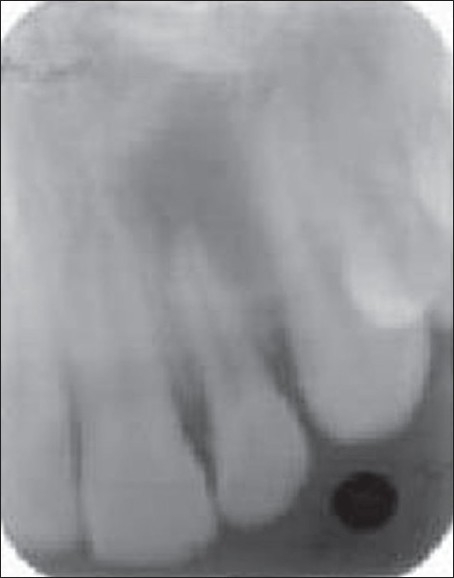
Preoperative

The patient was recalled the following day. Chemomechanical preparation was completed, and an intracanal medication of calcium hydroxide was given. In spite of repeated intracanal medication, the symptoms persisted and hence a periapical surgery was planned. Bilateral infra-alveolar block was administered, and a full-thickness mucoperiosteal flap was elevated. The lesion measuring 15×10 mm was curetted. The root canal was obturated with zinc oxide eugenol sealer and gutta-percha by lateral condensation. Root end resection was done. Retro-preparation was done using ultrasonic tips and was restored with type II glass ionomer cement. The periapical wound was flushed with saline. Biphasic calcium phosphate was mixed with saline to form a thick paste and carried to the periapex with an amalgam carrier and gently condensed into the periapical bone cavity. The flap was repositioned and sutured in place with a 3.0 black silk. An immediate postoperative radiograph was taken [Figure 1B]. Sutures were removed after 1 week. The patient was reviewed periodically. Density of the bone graft material decreased in size, as seen by the change in its radiopacity. On review at the end of 6 months, the bone graft was seen to be replaced with normal trabecular pattern of the bone [Figure 1C].

**Figure 1B:**
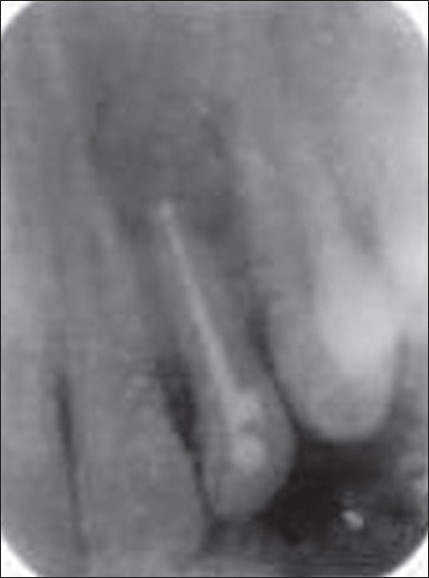
Postoperative

**Figure 1C:**
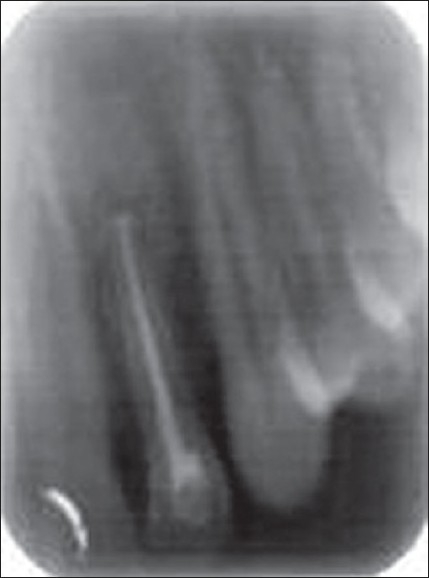
6 months' follow-up

**Figure 1D:**
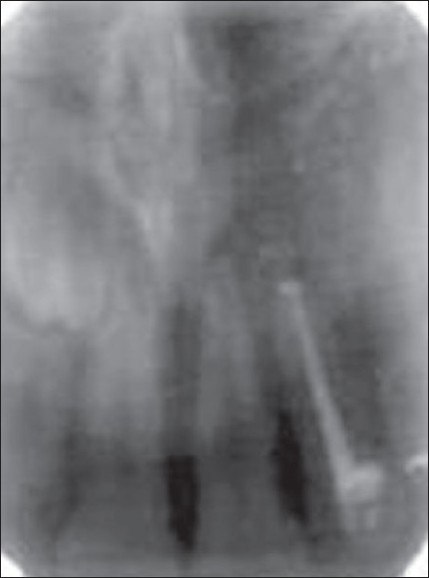
2 years' follow-up

Biphasic calcium phosphate was used in two other similar cases, which were followed for the treatment outcome [Figures 2A, 2B, 3A and 3B].

**Figure 2A:**
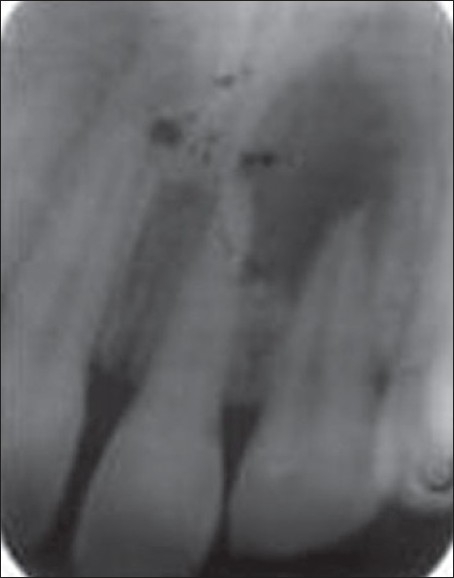
Preoperative

**Figure 2B:**
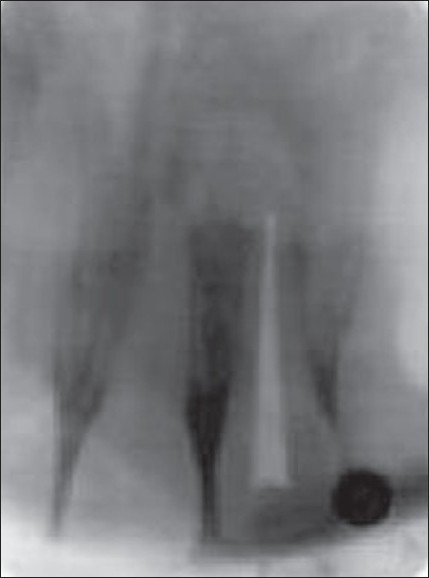
3 months' follow-up

**Figure 3A:**
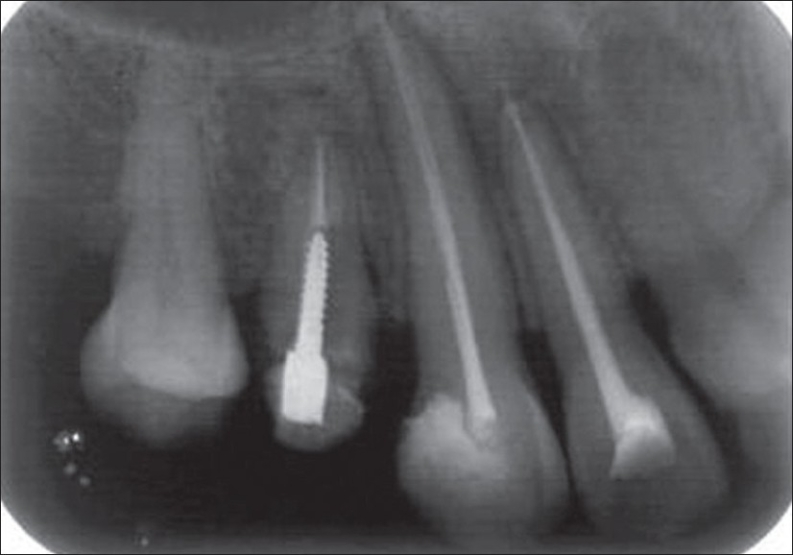
Preoperative

**Figure 3B:**
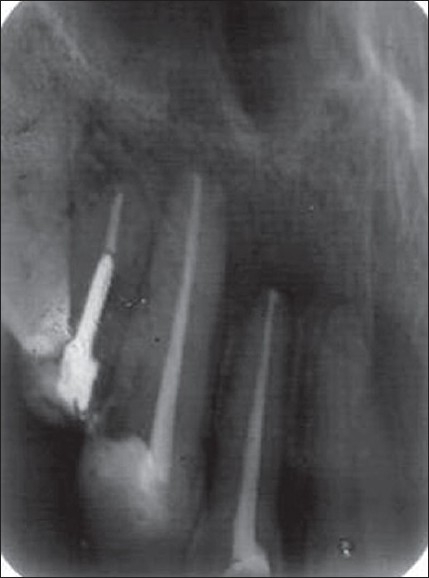
2 months' follow-up

## DISCUSSION

Repair can be defined as healing of an injured tissue that leads to the formation of a tissue that differs in morphology or function from the original tissue. Regeneration is used to describe a healing that leads to complete restoration of morphology and function. The recent trend is to find a biocompatible material to regenerate a diseased or damaged part of the body to its original status. Ceramics used for these purposes are termed “bioceramics.”[11]

Calcium phosphate-based bioceramics have been used in medicine and dentistry for nearly two decades. Applications include dental implants; percutaneous devices; and use in periodontal treatment, alveolar ridge augmentation, maxillofacial surgery, otolaryngology, orthopedics, and spinal surgery.[12] In this study, we have used the BCP ceramic for filling bone defects after periapical surgery.

There is necessity for replacing bone that has been lost due to disease or damage. Several bone substitutes are proposed for filling these defects. The use of graft materials in the bone defects prevents hematoma formation and acts as scaffold and promotes bone formation. The degradation of these bone substitutes is necessary as they are ultimately to be replaced by newly formed bone.[13] Resorbable ceramics act as temporary space fillers or scaffolds for new tissue to develop. Natural tissue reconstruction occurs simultaneously with resorption.[14]

The biphasic structure of BCP ceramics, characterized by different degradation rates for HA and β-TCP, accounts for their controlled bioactivity. BCP ceramics present intermediate degradation behavior, so that their progressive resorption and ultimate replacement are adapted to bone in growth.

Results of the previous study on tissue response to biphasic calcium phosphate ceramic with different ratios of HA/β-TCP in periodontal osseous defects have indicated that the ratio of HA to β-TCP in the ceramic implant influenced periodontal wound healing when used in the treatment of chronic osseous defects in dogs. Higher HA/β-TCP ratio tended to show greater gain in attachment level, accompanied by accelerated new bone formation as shown histologically. When β-TCP was removed from the ceramic component, using only 100% porous HA, the gain was found to be less than that of the groups that received ceramic containing β-TCP. This may be due to the low dissolution rate of porous HA. Since this study showed that the combination of HA and β-TCP has better new attachment level and bone regeneration, it would indicate that β-TCP plays an important role in cell proliferation, revascularization, and osteogenesis. However, when the opposite end of the ratio was examined, i.e., 0HA/100β-TCP, probing attachment level was also found to be less than that of the group that received the ceramic with HA. Based on histological results of the above study, 65HA/35β-TCP ratio appears to demonstrate greater gain in attachment level and bone regeneration.[15]

It has been hypothesized that the biphasic nature of this ceramic serves two basic functions: i) initiates cell growth and differentiation; and ii) acts as a scaffold for cell maturation and bone formation, i.e., having the property of osteoinductivity.[15]

Manjubala *et al.* studied the bone in-growth induced by biphasic calcium phosphate ceramic in femoral defect of dogs. BCP ceramic composed of 55% hydroxyapatite (HA) and 45% β-tricalcium phosphate was grafted in femoral defects of dogs. In this study, radiographic results showed that the process of ossification started after 4 weeks of grafting, and the defect was completely filled with new woven bone after 12 weeks. Histological examination of the tissue showed the formation of osteoblast inducing the osteogenesis in the defect. The collagenous fibrous matrix and the complete Haversian system were observed after 12 weeks. The blood serum was collected postoperatively, and biochemical assays for alkaline phosphatase activity were carried out. The measurement of alkaline phosphatase activity levels also correlated with the formation of osteoblast-like cells. This microwave-prepared BCP ceramic has proved to be a good biocompatible implant, as well as an osteoconductive and osteoinductive material to fill bone defects.[16]

The HA and β-TCP have been used in the same ratio in this study, prepared by the microwave method (material supplied by Indian Institute of Technology, Chennai). The postsurgical results, obtained both clinically and radiographically, showed predictable clinical outcome. Complete periapical healing was evident on the radiograph over a period of 2 years [Figure 1D]. Biphasic calcium phosphate shows potential regenerative properties and needs further exploration in clinical conditions.
